# Assessing the Accuracy and Completeness of OpenStreetMap Sidewalks: A Comparative Study With Google Street View

**DOI:** 10.5888/pcd23.260255

**Published:** 2026-10-01

**Authors:** Brianne L. Nichols, Caroline Groth, Tyler D. Quinn

**Affiliations:** 1 Department of Epidemiology and Biostatistics, West Virginia University School of Public Health, Morgantown, West Virginia

## Abstract

**Introduction:**

OpenStreetMap is an open-source and crowd-sourced geospatial database and one of the few available sources of sidewalk data for walkability research; however, its accuracy and completeness have not been systematically assessed. This study aimed to compare OpenStreetMap sidewalk data with Google Street View.

**Methods:**

We clipped an OpenStreetMap map of pedestrian-accessible roads in Maryland to 141 census tracts (~10.0% of census tracts in Maryland) using stratified random sampling across tertiles of population density. Fifty points within each census tract were randomly distributed across qualifying roads (n = 7,050). We determined sidewalk presence at each point in OpenStreetMap and Google Street View. We excluded points without Google Street View, resulting in a final sample of 6,103 points. In points with sidewalks in Google Street View (n = 3,674), logistic mixed effects models estimated odds of OpenStreetMap–Google Street View sidewalk agreement across population density tertiles and themes from the Centers for Disease Control and Prevention’s Social Vulnerability Index (SVI).

**Results:**

Of the total points (N = 6,103), 57.8% (3,530) indicated sidewalk agreement between OpenStreetMap and Google Street View. Agreement was 30.2% among points with sidewalks present in Google Street View. Among points with sidewalks (3,674) present in Google Street View, odds of sidewalk agreement were higher in mid-density tracts (odds ratio [OR], 2.43; 95% CI, 0.63–9.40) and high-density tracts (OR, 4.50, 95% CI, 1.16–17.38) than in low-density tracts. Tracts in which a racial and ethnic minority population was larger than the non-Hispanic White population had lower sidewalk agreement (OR, 0.11; 95% CI, 0.002–0.74); no other SVI theme was associated with agreement.

**Conclusion:**

While OpenStreetMap sidewalk data were generally accurate when available, the sidewalk data were incomplete, especially in areas with low population density or areas in which a non-Hispanic racial and ethnic minority population was larger than the White population.

**Summary**

**What is already known on this topic?**

OpenStreetMap is one of the few available sources of sidewalk data for walkability research, but little is known about the accuracy or completeness of OpenStreetMap sidewalk data.

**What is added by this report?**

OpenStreetMap sidewalk data were incomplete, especially in areas with low population density or with a high proportion of residents who are members of racial and ethnic minority populations.

**What are the implications for public health practice?**

OpenStreetMap sidewalk data are not complete enough to be a data source for walkability research or public health planning.

## Introduction

Neighborhood walkability is consistently associated with higher levels of physical activity (1–3). Walkability is commonly assessed by using surveys, environmental audits, or geographic information systems (GIS) (4) Survey- and audit-based measures frequently capture data on sidewalk presence and condition (5); for example, widely used instruments such as the Neighborhood Environmental Walkability Scale (6) and the Physical Activity Neighborhood Environment Survey (PANES) (7) include multiple items on sidewalk completeness and maintenance. Studies examining individual built environmental features typically report positive associations between the presence of sidewalks and physical activity (1,8).

In contrast, GIS-based measures of walkability rarely incorporate sidewalk data (4,9,10), likely due to limited data availability at the necessary spatial scale (4). This discrepancy between GIS measures and survey or audit measures may lead to inconsistencies across studies. Additionally, because audits are time intensive and less common (4), most studies that include sidewalks as a component of walkability rely on self-report, which may introduce measurement error. Previous research suggested that self-reported environmental measures often deviated from GIS-based measures (11). For example, a study comparing Neighborhood Environment Walkability Scale responses with GIS-based measures found low to moderate correlations across multiple domains, with some constructs having no significant correlation (12); PANES performed similarly (13).

OpenStreetMap, a source of volunteered geographic information, provides publicly available sidewalk data. However, the completeness and accuracy of these data remain largely unverified. Prior research evaluating other OpenStreetMap feature types (eg, overall land coverage, building footprints) identified substantial differences in data quality by geographic location and feature class (14,15), but sidewalks have not been examined.

To address this gap, we aimed to evaluate the completeness and accuracy of OpenStreetMap sidewalk data across Maryland. We hypothesized that data accuracy and completeness would be lower in more rural and lower-income areas. We sought to assess the utility of OpenStreetMap as a source of sidewalk data for walkability and physical activity research.

## Methods

### Sample

We selected Maryland as the study area due to its diversity in racial and ethnic composition, rural–urban mix, and manageable number of census tracts. We obtained data on census tract boundaries and 2020 population from the US Census Bureau (16). We excluded census tracts with no reported population from the sample, which resulted in 1,467 tracts. We calculated population density (population per square mile) and grouped tracts into tertiles (high-, mid-, low-density), with 489 tracts in each tertile. From each population density tertile, we randomly selected 47 tracts, yielding a final sample of 141 tracts (~10% census tracts).

### Sidewalk selection and review process

To begin, we created a base layer of qualifying OpenStreetMap roads for each of the selected census tracts in QGIS (QGIS). OSM roads were selected for the base layer, rather than Google Street View (GSV) images, to better record bias which will be introduced due to lack of GSV images in rural areas. Roads were considered qualifying if they were publicly accessible and open to pedestrians, as defined by OpenStreetMap’s highway classification system (17). We excluded roads classified as motorways, service roads, and link roads due to their limited pedestrian accessibility. Qualifying roads included trunk roads, primary roads, secondary roads, tertiary roads, unclassified roads, and residential roads. The qualifying road layer was saved as a shapefile and imported to ArcGIS Pro (Esri) for all future spatial data steps.

We distributed 50 points randomly across the created road network (N = 7,050 points). After we created points, we evaluated the presence or absence of adjacent sidewalks to each point on both OpenStreetMap and the corresponding Google Street View images. We reviewed Google Street View and OpenStreetMap data. We first verified that each point in OpenStreetMap matched the corresponding location in Google Street View, which provided our “ground truth” by comparing road names and surrounding landmarks. We examined each point by visually inspecting the OpenStreetMap map and Google Street View images to record the presence or absence of adjacent sidewalks on both sides of the road. All points were reviewed by 2 independent reviewers (S.T., E.T., B.N.). Using a purpose-built data collection form in Microsoft Excel, reviewers recorded whether a sidewalk appeared on one, both, or neither side of the street in Google Street View and OpenStreetMap. If a point was located at an intersection, reviewers ensured consistency by using the same orientation for sidewalk coding in both OpenStreetMap and Google Street View. If a road segment was present in OpenStreetMap but missing in Google Street View, reviewers recorded this discrepancy. A third reviewer (B.N.) examined discordant or unclear determinations and corrected them when necessary. Finally, the date of the available Google Street View images and the OpenStreetMap tag data, which included additional descriptive information about OpenStreetMap features in that area, were collected at each point. We noted OpenStreetMap tags of road features indicating a sidewalk.

### Calculations of sidewalk agreement

After the review process, we created the primary outcome for all analyses as binary point-level agreement between OpenStreetMap and Google Street View sidewalk data (1 = agreement or 0 = disagreement). We defined agreement as 1 side of the street or both sides of the street having sidewalk data agreement between OpenStreetMap and Google Street View and disagreement as neither side of the street having sidewalk data agreement between OpenStreetMap and Google Street View. While we recorded more complex data, including partial agreement (ie, agreement on 1 side of the street and disagreement on the other side), we used the binary agreement defined above to allow for practical interpretation and logistic statistical modeling for all analyses. We excluded points without Google Street View images from calculations of point-level agreement.

### Social factors

Social factors were represented by themes from the Centers for Disease Control and Prevention’s Social Vulnerability Index (SVI) for each census tract (18). The SVI includes information across 4 themes: Theme 1 (socioeconomic status), Theme 2 (household characteristics), Theme 3 (race and ethnicity), and Theme 4 (housing type and transportation). Each theme is a percentile ranking, with 1 indicating the highest social vulnerability and 0 indicating the lowest social vulnerability. In addition to the 4 themes, we included an overall percentile ranking of social vulnerability in an individual model.

We assessed the normality of continuous variables by using the Shapiro–Wilk test; non-normally distributed variables are reported as the median (interquartile range [IQR]). In addition to variables from the SVI, population density was reported as the median number of residents per square mile within each density tertile. We summarized Google Street View imagery dates as the median (IQR) of the reported dates for all collected points within each census tract density tertile.

### Analytic approach

We summarized binary sidewalk agreement between OpenStreetMap and Google Street View descriptively as percentage agreement across all sampled points and a subsample of points with sidewalks in Google Street View. This study used ArcGIS Pro to generate a map depicting agreement between GSV and OSM sidewalk data. Jenks natural breaks, a method to maximize separation between data groupings, classified sidewalk agreement into three categories. Logistic mixed effects models examined the odds of sidewalk agreement across population density tertiles with and used low-density tertile as the reference. Additional separate logistic mixed effects models examined the odds of sidewalk agreement across each of the 4 SVI themes and total SVI as the primary predictors, treated continuously. We accounted for spatial autocorrelation (a violation of the assumption of independence based on closer geographic space having more closely related values) (19) between points by using census tract geographic identifiers modeled as random effects.

We excluded several census tracts from the logistic models. First, we excluded 11 census tracts that had no sidewalks present in Google Street View at any of the observed points. We made this exclusion after considering the descriptive statistics because examining OpenStreetMap completeness was the primary aim of the models. Second, we excluded census tracts with fewer than 5 total valid points to allow model convergence, creating a final sample size of 3,661 points from 122 tracts for the logistic models. All statistical analyses were performed in RStudio (Posit Team).

## Results

Median population density was 517 persons per square mile in low-density tracts, 3,189 in mid-density tracts, and 7,810 in high-density tracts (Table 1). Percentiles for overall SVI ranged from 0.0014 to 0.9788. Higher-density tracts were generally more socioeconomically disadvantaged and had greater racial and ethnic diversity, a greater proportion of households without vehicle access, and higher rates of residential crowding.

**Table 1:** Characteristics and Selected SVI^a^ Variables of Included Census Tracts by Tertile of Population Density

Variable^b^	Median (IQR)		
	Low-density	Mid-density	High-density
No. of census tracts	47	47	47
Google Street View image date, MM/YY	10/22 (07/21–07/23)	08/22 (08/21–04/23)	08/22 (05/22–01/23)
Population density, persons/square mile	517 (179–982)	3,189 (2,507–4,315)	7,810 (6,645–10,669)
**SVI theme 1: Socioeconomic status, %**			
<150% of federal poverty level	6.5 (4.0–11.7)	10.3 (5.9–21.0)	19.1 (13.7–25.6)
Unemployed	4.4 (2.7–5.7)	4.1 (2.7–6.6)	5.3 (4.2–8.5)
Without high school diploma	6.1 (3.6–9.7)	8.0 (3.4–10.9)	11.8 (7.8–17.9)
**SVI theme 2: Household characteristics, %**			
Aged ≥65 y	17.3 (14.1–20.9)	15.8 (11.9–21.0)	14.3 (12.7–16.2)
Aged ≤17 y	21.7 (17.9–22.0)	21.4 (18.4–25.9)	23.6 (18.7–27.0)
Single-parent household	3.4 (1.8–5.5)	5.0 (2.8–8.1)	5.9 (3.6–12.1)
**SVI theme 3: Race and ethnicity, %**			
Hispanic	4.1 (1.3–8.2)	6.4 (3.4–12.3)	7.3 (2.8–24.4)
Non-Hispanic Asian	2.0 (0.8–4.9)	1.8 (0.9–6.0)	2.4 (0.9–7.4)
Non-Hispanic Black	6.5 (3.3–17.2)	17.6 (6.9–56.1)	34.5 (12.0–79.2)
**SVI theme 4: Housing type and transportation**, %			
Households without car	2.8 (1.3–6.1)	4.7 (1.6–11.5)	9.9 (5.3–20.9)
Housing units with more people than rooms	0.5 (0–1.5)	0.7 (0–2.9)	2.4 (0.3–4.5)

Abbreviation: SVI, Social Vulnerability Index; IQR, interquartile range.

a The SVI was developed by the Centers for Disease Control and Prevention (18).

b All variables are presented as median (IQR) because they were determined to be nonparametric via Shapiro–Wilk tests. All SVI values are the median percentage within each population-density tertile and therefore will not sum within tertiles to 100%.

### Google Street View coverage

Of the 7,050 points reviewed across 141 sampled census tracts, Google Street View was available in 6,103 (86.6%); 947 points lacked Google Street View imagery and were excluded from all subsequent analyses. Notably, 72.8% (n = 689) of the unavailable points were located in the low-density tertile, suggesting a spatial disparity in Google Street View coverage. The median Google Street View image date of included points was 2022; we observed no significant differences between population density tertiles.

### Sidewalk presence and agreement

Among the points with available Google Street View imagery, 3,674 points (60.2%) had sidewalks on at least 1 side of the street and were retained for the final analysis. Points of nonagreement tended to occur when OpenStreetMap lacked data rather than as a result of inaccurately added data. We found only 11 points where OpenStreetMap indicated the presence of a sidewalk that was not indicated in Google Street View; we found 2,866 points where Google Street View indicated the presence of a sidewalk that was not indicated in OpenStreetMap (Figure 1).

**Figure 1:**
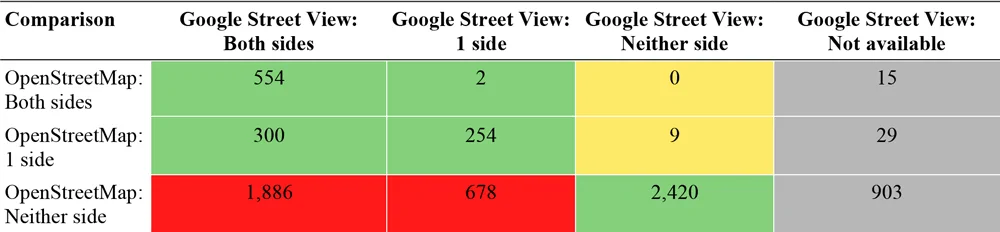
Point-level agreement between Google Street View and OpenStreetMap. Green cells indicate concordant pairs; yellow cells indicate discordant pairs, denoting inaccuracy in OpenStreetMap; red cells indicate discordant pairs, denoting incompleteness in OpenStreetMap; and gray cells indicate that data were not available in Google Street View. Only the points in “Google Street View: Both sides” and “Google Street View: 1 side” were used in logistic mixed models.

**Table:** 

Comparison	Google Street View: Both sides	Google Street View: 1 side	Google Street View: Neither side	Google Street View: Not available
OpenStreetMap: Both sides	554	2	0	15
OpenStreetMap: 1 side	300	254	9	29
OpenStreetMap: Neither side	1,886	678	2,420	903

### Descriptive statistics of sidewalk agreement

The overall agreement for all points was 57.8%, with 78.3% agreement in the lowest density tertile, 52.9% in the mid-density tertile, and 47.5% in the highest density tertile (Table 2). Agreement for points with sidewalks present in Google Street View was 30.2% overall, 19.7% for the lowest density tertile, 26.9% for the mid-density tertile, and 35.4% for the highest density tertile. Including OpenStreetMap tag data as an additional identifier of sidewalks slightly increased agreement among points with sidewalks. However, tag data were more likely to incorrectly code a sidewalk as being present in OpenStreetMap when it was not present in Google Street View, resulting in the overall point agreement not changing. 

**Table 2:** Percentage Sidewalk Agreement^a^ Between OpenStreetMap and Google Street View Overall and by Tertile of Population Density

Agreement	All observed points with Google Street View imagery available, % (no./total)	Only points with sidewalks present in Google Street View, % (no./total)
**Sidewalk agreement**		
Overall agreement	57.8 (3,530/6,103)	30.2 (1,110/3,674)
Overall agreement including OpenStreetMap tags	57.7 (3,520/6,103)	32.9 (1,210/3,674)
**Population density tertile**		
Low-density	78.3 (1,300/1,661)	19.7 (88/447)
Mid-density	52.9 (1,174/2,220)	26.9 (384/1,426)
High-density	47.5 (1,056/2,222)	35.4 (638/1,801)

a Percentage agreement between points, where agreement is defined as either 1) neither OpenStreetMap nor Google Street View show a sidewalk, or 2) both OpenStreetMap and Google Street View have at least 1 sidewalk.

Figure 2 shows percent agreement between GSV and OSM sidewalks for census tracts included in this study. Tracts with no GSV images tend to be larger, and therefore very rural; while tracts with greater than 60% agreement are typically located near major urban areas (outside Washington, DC, or Baltimore, MD). 

**Figure 2:**
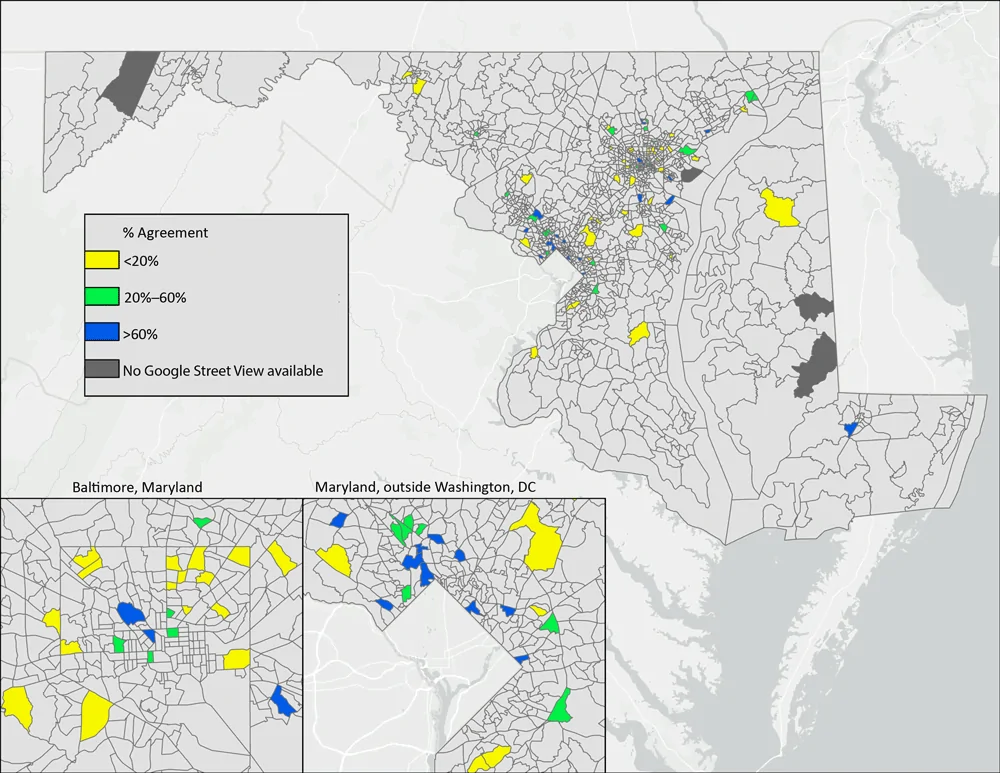
Agreement in presence of sidewalks between OpenStreetMap and Google Street View for randomly selected census tracts, categorized by Jenks natural breaks, in Maryland.

### Statistical modeling of sidewalk agreement

High-density tracts had significantly higher odds of agreement compared with low-density tracts (odds ratio [OR], 4.50; 95% CI, 1.16–17.38; *P* = .03) (Table 3). The odds of sidewalk agreement did not differ significantly between mid- and low-density tracts (OR, 2.43; 95% CI, 0.63–9.40; *P* = .20). In SVI models, only Theme 3, race and ethnicity, was significantly associated with sidewalk agreement, such that for every 1 percentile higher racial and ethnic minority residency within a census tract, we found 89% lower odds of agreement in sidewalk presence (OR, 0.11; 95% CI, 0.002–0.74; *P* = .02). We found no significant association between overall SVI score and sidewalk agreement (OR, 0.65; 95% CI, 0.12–3.59; *P* = .62) or any other SVI theme.

**Table 3:** Odds of Sidewalk Agreement Between OpenStreetMap and Google Street View, by Tertile of Population Density^a^ and SVI Theme^b^

Variable	Odds ratio (95% CI)	*P* value
**Population density tertile**		
Low-density	1 [Reference]	—
Mid-density	2.43 (0.63–9.40)	.20
High-density	4.50 (1.16–17.38)^b^	.03
**SVI**		
Total SVI	0.65 (0.12–3.59)	.62
Theme 1: Socioeconomic status	0.45 (0.08–2.54)	.37
Theme 2: Household characteristics	0.55 (0.08–3.78)	.54
Theme 3: Race and ethnicity	0.11 (0.002–0.74)	.02
Theme 4: Housing type and transportation	2.29 (0.41–12.68)	.34

Abbreviation: SVI, Social Vulnerability Index.

a Mixed effects logistic models for population density show odds of binary agreement across population density tertiles. Logistic mixed effects models for the SVI are separate models for the odds of binary sidewalk agreement within each census tract for the total SVI and each SVI theme. All models completed on a subsample of data were restricted to tracts with sidewalks present in Google Street View and with at least 5 available points for review.

b The SVI was developed by the Centers for Disease Control and Prevention (18).

## Discussion

This methodological study assessed the accuracy and completeness of OpenStreetMap sidewalk data using Google Street View imagery as ground truth across Maryland. OpenStreetMap showed high accuracy, with few false positives, but low overall completeness. Completeness was higher in more densely populated areas but lower in areas with greater racial and ethnic diversity. While OpenStreetMap has potential as a sidewalk data source, it should be used with caution.

To our knowledge, this is the first study to directly assess the accuracy and completeness of OpenStreetMap sidewalk data. Prior validation studies across other built environmental feature types showed substantial variability by geography, feature, and evaluation method. A study examining data quality in France found a large variation in data quality depending on map features and data quality metrics (20). A 2022 study, conducted in 5 counties in Poland and focusing on basic land-cover features, reported completeness ranging from 82% to 51% (14). Another study, conducted in Munich, Germany, and assessing the completeness of building footprints, found that OpenStreetMap data covered only 66.1% of buildings, compared with their ground truth (15). Similarly, a study in Italy reported regional differences in building footprint completeness; urban areas had 59% surface area coverage and rural areas only 47% (21). A 2023 study examining the food environment points-of-interest across 5 European countries found high accuracy but limited completeness (22). These findings of these studies align with our results, suggesting that OpenStreetMap data are often accurate but incomplete. Sidewalk data may present additional challenges due to limited external data sources, which may increase reliance on local knowledge for contributions. This may explain the lower completeness observed in this study (30.2%), as compared with other feature types in previous studies (14,15,20).

Although the accuracy of OpenStreetMap sidewalk data was high, agreement between OpenStreetMap and Google Street View sidewalk data was low, indicating incomplete OpenStreetMap coverage. Discordant pairs were more common when Google Street View identified sidewalks, suggesting OpenStreetMap frequently misses infrastructure. This finding is reflected in low sensitivity (30.2%) and negative predictive value (48.6%), alongside high specificity (99.6%) and positive predictive value (99.2%) when using Google Street View as the ground truth. High specificity indicates OpenStreetMap accurately classified the absence of sidewalks, while low sensitivity indicates poor detection of sidewalk presence. Of the 9 false-positive points (ie, those with reported sidewalks in OpenStreetMap but not in Google Street View), 2 points were from the low-, 4 from the mid-, and 3 from the high-density tertiles. Both points in the low-density and 2 of the 3 points in the high-density tertiles were from the same census tract. In the high-density tract, these points represented the same misreported length of sidewalk, while in the low-density track, these points were geographically disparate. These findings suggest that although OpenStreetMap data in our sample were accurate when features are present, they were incomplete and may be insufficient for health research or urban planning applications where decisions inform public health or financial investments.

Agreement was higher across all points than among locations with sidewalks in Google Street View, particularly in lower-density areas. This pattern was caused by the high prevalence of “no sidewalk” observations in both datasets. Because OpenStreetMap relies on manual mapping, areas with fewer sidewalks tended to show higher agreement by default, indicating both true absence in Google Street View and missing data in OpenStreetMap. Additionally, sidewalks were more common in higher-density areas.

Among points with sidewalks present in Google Street View, both percentage agreement and odds of agreement between OpenStreetMap and Google Street View increased with population density. This finding likely reflects greater mapping activity in higher-density areas due to increased engagement by individual mappers and organizations. Although most points without Google Street View (72.7%) were in the low-density tertile, their inclusion would likely not meaningfully improve completeness, as missingness was primarily caused by incomplete OpenStreetMap sidewalk data. Among points without Google Street View, only 2.2% indicated sidewalks in the low-density tertile, compared with 10.7% and 11.7% in the mid- and high-density tertiles, respectively.

Urban areas not only had greater sidewalk agreement in OpenStreetMap but also greater sidewalk coverage in Google Street View. This finding may reflect transportation code that mandates that specific types of new construction in urban areas will include sidewalks (23). No such regulations exist in rural areas; rather, those building new construction in rural areas are encouraged to include “multipurpose shoulders” (24).

Of the 4 SVI themes, only race and ethnicity was significant. Census tracts with higher percentages of non-White residents had significantly lower odds of agreement between OpenStreetMap and Google Street View. While population density and the SVI theme of race and ethnicity are correlated, the directionality of the association would introduce a bias towards the null, suggesting the actual association is slightly stronger. Given that disagreement is likely caused by missing sidewalks in OpenStreetMap, this finding suggests greater data incompleteness in more racially and ethnically diverse areas. This pattern may reflect contributor bias (25); OpenStreetMap mappers are predominantly White and tend to map areas near where they live or travel, with most contributions produced by a small group of highly active users (26,27).

The association may instead reflect socioeconomic and geographic factors rather than race and ethnicity alone. In our sample, low-income, less racially diverse tracts were largely rural, where sidewalks are less common and Google Street View coverage is limited. Most excluded tracts and missing Google Street View data occurred in low-density areas, likely skewing the analytic sample and potentially masking associations with socioeconomic status. Prior work shows mappers concentrate on urban, wealthier areas, while those mapping rural or poorer areas typically make fewer contributions (28). Regardless of mechanism, uneven OpenStreetMap coverage raises concerns about data equity, as gaps in completeness may bias research and contribute to inequities in planning and resource allocation (29).

### Strengths and limitations

A key strength of this study is its novel, generalizable assessment of OpenStreetMap accuracy and completeness. Manual review of Google Street View as ground truth enhanced internal validity. While Maryland’s diverse mix of rural and urban areas strengthens the generalizability of the findings to other locations, replication in other states or countries is still necessary.

Limitations include data availability; 13.4% of reviewed points were not available in Google Street View, 72.8% of which were in low-density tracks, resulting in underrepresentation of those areas in the SVI analyses. We did not assess sidewalk quality; sidewalks were classified based on their apparent walkability, and no OpenStreetMap sidewalks were contradicted by Google Street View due to sidewalk quality. We recorded the date of Google Street View data; however, we did not use this information to exclude data. We used the most recent data available. However, approximately 8% of available points were more than 10 years old, with 10%, 9%, and 6% of points being more than 10 years old in low-, mid-, and high-density census tracts, respectively. While outdated Google Street View may introduce inaccuracies, the influence on our results was likely minimal. Outdated Google Street View images would primarily introduce discordance with OpenStreetMap if sidewalks were removed, which is rare. Furthermore, most discrepancies where sidewalks appeared in OpenStreetMap but not Google Street View had imagery from the last 5 years. OSM dates are not recorded with other feature data and documentation; instead, OSM history for any geographic extent is recorded and stored separately. The goal of OSM is to provide up to date geographic information and practical utilization would treat OSM data as current, therefore OSM dates were not considered to reflect real world use and for study feasibility. The lack of accessible data is a potential concern for researchers, but outside the scope of this study. We modeled points as binary agreement, rather than categorical agreement, which would have captured points where OpenStreetMap inaccurately reported nonexistent sidewalks due to model convergence issues based on small cell size. Finally, models assumed linearity; future work could explore spline-based associations between sidewalk completeness and demographic characteristics.

### Practical implications and future directions

We informally noted that fragmented sidewalks appeared less likely to be mapped in OpenStreetMap than those within larger networks. Connecting these fragments could improve walkability at lower cost than building new infrastructure; however, if OpenStreetMap data guide resource allocation, the potential for such high-impact, low-cost investments might be overlooked.

Future work should assess accuracy and completeness of OpenStreetMap data across larger geographic areas and other map features. Future resources should also be allocated to improve the completeness of open-source and crowd-sourced geospatial data such as OpenStreetMap. Some areas that have invested in mapping, including through map-a-thons, may have greater data completeness. However, the variability of map completeness is a barrier to widespread public health use.

### Conclusion

OpenStreetMap in general and the sidewalk data specifically have the potential to fill a gap in spatial data availability with high utility for walkability and research into physical activity. The accuracy of the sidewalk data observed provides an encouraging sign of the potential utility in open-source and crowd-sourced geospatial data. However, due to the lack of data completeness as demonstrated in this study, caution should be taken when using sidewalk data, especially for high-impact public health decisions.
